# 循环肿瘤细胞在早期肺癌诊疗中的研究进展

**DOI:** 10.3779/j.issn.1009-3419.2017.10.07

**Published:** 2017-10-20

**Authors:** 新春 段, 志东 刘, 绍发 许

**Affiliations:** 101149 北京，首都医科大学附属北京胸科医院胸外二科 Department of Toracic Surgery, Beijing Chest Hospital, Capital Medical University, Beijing 101149, China

**Keywords:** 循环肿瘤细胞, 液体活检, 肺小结节, 肺肿瘤, 诊断, Circulating tumor cells, Liquid biopsy, Small pulmonary nodules, Lung neoplasm, Diagnosis

## Abstract

循环肿瘤细胞（circulating tumor cells, CTCs）作为液体活检的一种重要类型，在肺癌的筛查诊断、疗效评估、术后监测与预后判断等方面显示出越来越丰富的临床价值。随着对肺癌高危人群筛查工作的进展，大量肺小结节患者被检出，但是肺小结节不等于肺癌，而且据统计良性比例达90%-95%，这使得该部分患者在首次就诊时的良恶性鉴别诊断成为临床医生面临着的新的机遇与挑战。CTCs检测技术的不断进步与完善，是否可以在早期肺癌的鉴别诊断中发挥更大的作用，此外，它是否对早期肺癌手术治疗时的操作具有指导意义，这还需要进一步科研探索，以期将来实现临床转化。

## Abstract (en)

As one of the important liquid biopsies, circulating tumor cells (CTCs) shows more and more clinical values in the treatment of lung cancer such as diagnostic screening, treatment evaluation, postoperative monitoring, and prognosis predicting etc. A large number of small pulmonary nodules patients are detected when screening the high risk population of lung cancer. However, small lung nodules are not equal to lung cancer, and 90%-95% of them are benign lesion, therefore, to accurately and correctly differentiate whether it is benign or malignant when patients firstly detected and treat a small pulmonary nodule is become a new opportunity and challenge for clinician. With the improvement of CTCs detection technology, whether it will play an important role in early differential diagnosis of lung cancer. And whether it will have clinical significance to early lung cancer surgery operations. These require further researches and explorations so as to achieve clinical transformation in the future.

世界范围内，肺癌发病率长期以来居高不下，而且保持成为肿瘤患者死亡的主要原因^[1, 2]^。国内亦然，无论乡村城市、无论男女，肺癌的发病率和死亡率均排在首位，成为影响社会公众健康的重大问题^[3]^。肺癌预后极差，而且70%-75%的患者发现时已处在晚期，失去了手术机会。为此，2011年美国率先开展了国家肺癌筛查试验（National Lung Screening Trial, NLST），发现低剂量螺旋计算机断层扫描（low-dose computed tomography, LDCT）可以提高早期肺癌患者的检出率，进而显著降低了肺癌患者的死亡率^[4]^。

随着国内肺癌筛查指南的制定^[5, 6]^，公众健康意识的提高，进行LDCT筛查群体日益增加，结果是大量肺小结节的患者被发现、被检出。但是，肺小结节并不等同于肺癌，相反，90%-95%的病例为良性^[4]^。为了科学合理地处理该部分患者，多个国家分别制定了肺小结节处理指南^[7-9]^。此外，医院设立多学科团队（Multidisciplinary Team, MDT），主要以胸外科、影像科、呼吸科与肿瘤内科等科室组成肺小结节诊疗中心，共同提供诊疗方案。在这过程中，医生常常依据肺小结节的大小、密度、形态、成分比例、以及特殊征象来判断其良恶性，对高度怀疑肺癌的患者建议PET-CT检查、穿刺或者直接手术治疗；对难以判断性质的常依据情况嘱咐患者进行3个月-12个月随访复查。时间被认为是最好的诊断，然而这些处理策略常常造成过度诊疗或者延误治疗，让患者等待复查的同时加剧了患者焦虑心理，反复复查也增加了社会经济负担^[10]^。因此，精准地鉴定肺小结节良恶性成为临床面临着的新的机遇与挑战。

1869年，澳大利亚Ashworth^[11]^医生首次提出了CTC的概念。20世纪90年代，人类开始了对CTCs的科研探索，由于检测技术的限制，早期大部分研究局限在中、晚期肿瘤。但理论上讲，超过2 mm的肿瘤便可诱导血管生成，为肿瘤细胞入血提供基础，有研究^[12, 13]^通过动物实验验证了这点，即在肿瘤早期便可在外周血中检测出CTCs。随着检测技术的不断进步，CTCs检出的敏感性和特异性也随之提高^[14-17]^，因此，CTCs本身的检测，或者联合肿瘤标志物辅助影像学检查有望提高肺小结节良恶性鉴别诊断的准确率。本文主要针对CTCs，以及其在早期肺癌诊疗方面作一综述。

## 1 CTCs的概述

CTCs是指原发肿瘤或转移病灶内的肿瘤细胞通过主动迁移、侵袭或者外在因素干扰导致其被动脱落，从而进入循环血液所形成^[18, 19]^。有研究^[20]^表明，1 g肿瘤组织，大约有100万个肿瘤细胞，便可每日播散肿瘤细胞入血。但是，CTCs的数量在循环血液中常呈现出动态递减的特点，到达外周血时已十分稀少。据研究，每10^6^个-10^7^个白细胞中才可以检测到1个肿瘤细胞^[21, 22]^。理论上讲，它可以以单个细胞或者以群体细胞组成团块（circulating tumor microemboli, CTM）^[23]^进入循环血液，而进入的多少、快慢应主要由肿瘤本身的生物学特性决定，总之，肿瘤细胞进入循环血液是肿瘤本身生存进展的必然结果。

## 2 CTCs的生物学活性

当病灶中的肿瘤细胞获得迁移、侵袭的条件与机会时，会主动地下调上皮表型如EpCAM和E-cadherin，上调间叶表型如波形蛋白，通过上皮-间叶转换（epithelial mesenchymal transitions, EMT）、细胞骨架和细胞间连接紧密程度的改变以增加自身变形、运动的能力，以便顺利通过血管^[24, 25]^及淋巴管上皮细胞。进入循环血液的CTCs可表现为以下形式：①凋亡或者坏死。研究^[26]^表明虽然成千上万的肿瘤细胞进入循环血液，最终仅仅极少量可以到达远处脏器。在血流运行的过程中，大量CTCs受到物理剪切力、机械撞击、及免疫系统攻击^[27]^等诸多不利因素从而导致自身凋亡、坏死，同时崩解出ctDNA、RNA^[28, 29]^等其他物质。②存活。虽然CTCs在循环血液中存活时间短，有研究显示几小时至24 h不等，但是极少部分CTCs可以存活下来，最终迁移到第二脏器，如在骨髓内形成播散的肿瘤细胞（disseminated tumor cells, DTC）^[30]^。存活的机制是复杂的，CTCs为了存活，在循环中通过一系列机制去避免自身损毁，如聚集成癌栓避免物理因素带来的破坏、上调CD47逃逸免疫监视^[27]^、结合活化的血小板以利于粘附血管内皮细胞，为再次跨越血管内皮创造条件^[31]^、通过β-globin高表达增强CTCs抗氧化应激的能力^[32]^等等。③转移。肿瘤细胞的长期生存需要适宜其生长的微环境，稳定的外周环境是其生存的第一要素。CTCs经过细小的毛细血管时，血流速度缓慢，此时它们可经过间叶-上皮转化（mesenchymal-to-epithelial transition, MET），或者受到局部炎症、趋化因子、细胞因子以及黏附分子上调等^[33]^的影响再次实现跨血管内皮细胞迁移^[34]^，到达组织器官。骨髓是最常见最容易的定居部位^[35]^，也因此被称为DTC的“存储库”。定居后的CTCs可能在迁移的过程中失去了某些功能，暂不能分化增值，停留在静止状态，称为临床休眠肿瘤细胞，这一状态可持续10年以上^[36, 37]^。休眠的肿瘤细胞呈现异质性的特点，有干性非干性、表型差异之分，其中具有干性的、或中间表型的易于生长增殖^[38, 39]^。这期间它们中的一部分可逃逸免疫清除、抗肿瘤治疗^[40]^，直至自身主动创造再次增值分化的微环境或条件，或者先被动接受促癌因素刺激后再经自身主动增值分化，直到形成影像学可以识别出的微小转移灶。

## 3 CTCs的检测

CTCs在外周血中十分稀少，要从数以千万计以上背景的细胞中精准地检测到它们并非易事，这也一直是CTCs科研探索中的瓶颈所在，也是限制其在临床上发挥作用的主要原因。

### 3.1 外周血的富集分离

这点通常基于细胞的物理学特性和生物学特性^[41]^，前者是依据CTCs和正常细胞在大小、密度、电荷等方面的差异设计的，经典的方法如密度梯度离心法^[42]^、膜过滤法^[43]^（isolation by size epithelial tumor cells, ISET）、红细胞变形性富集法^[44]^、红细胞电学特征富集法^[45]^、3D微型过滤器^[46]^等；后者基于CTCs和正常细胞表面蛋白表达的差异性，利用抗原抗体结合原理所设计，如免疫磁性分离技术法^[47]^（immunomagnetic separation, IMS）、微流体芯片^[48]^分离技术法。

### 3.2 检测富集分离好的样本

该步骤主要基于CTCs细胞表面特异性表达分子标记物，采用细胞计数或者核酸检测的方法，比如CellSearch系统法^[47]^、配体靶向PCR法^[49]^（ligand-targeted PCR method, LT-PCR）、插入人端粒酶启动子和绿色荧光蛋白基因的单纯疱疹病毒法（virus trace method, oHSV1-hTERT-GFP）^[50]^、纳米检测法^[51]^、流式细胞术^[52]^（ﬂow cytometry, FCM）、逆转录-聚合酶链反应法^[53]^（reversetranscriptase PCR, RT-PCR）等方法。下面主要简述前三种方法：①CellSearch系统检测是美国早期发明的一项结合富集分离与检测为一体，自动化程度较高的技术。这项技术获得了美国食品药品管理局（Food and Drug Administration, FDA）认证，并应用于临床转移性乳腺癌、结直肠癌、前列腺癌CTCs的检测^[54]^。该技术首先利用肿瘤细胞为上皮细胞这一特性，以包被在磁珠上的上皮细胞粘附分子（epithelial cell adhesion molecule, EpCAM）抗体与肿瘤细胞表面抗原结合进行富集，其次对富集的样本进行免疫荧光标记（DAPI, CK-PE, CD45），最后，筛选出来的细胞中EpCAM(+)DAPI (+)CK-PE(+)CD45(-)为肿瘤细胞。此法虽然广泛应用，也获得许多国家的认可，但是在实践检验中发现其受到EMT效应影响，灵敏性、特异性低成为美中不足之处，尤其在早期肺癌诊断方面。②与CellSearch系统法相对应，国内一项原创检测技术LT-PCR法在肺癌鉴别诊断中具有较高的灵敏性和特异性^[17, 55]^，其试剂盒也已通过了中国国家食品药品监督管理局（China Food and Drug Administration, CFDA）的批准上市。该技术原理是基于肿瘤细胞表面表达某种特异性抗体，例如肺癌细胞表面会过量表达叶酸受体（folate receptor, FR），然后以配体类似物交联核苷酸片段作为检测探针，与肿瘤细胞表面受体靶向结合，从而将CTCs的数量转变为探针的数量，最后通过检测探针的PCR，计算出CTCs的数量。③oHSV1-hTERT-GFP法是国内最新，并拥有自主知识产权的一项CTCs检测技术，基本原理是依据肿瘤细胞端粒酶高启动活性，和溶瘤病毒——单纯疱疹病毒-1（herpes simplex virus-1, HSV-1）嗜肿瘤特性相结合，将端粒酶启动子和绿色荧光蛋白（green ﬂuorescent protein, GFP）基因插入HSV-1，以HSV-1为载体识别并感染肿瘤细胞，再通过端粒酶启动子特异启动基因表达绿色荧光蛋白，最后用流式细胞术检测和分选CTCs。此法已在6种实体器官肿瘤和淋巴瘤中检测，显示出了良好的灵敏性和特异性^[50]^。此法优势在于其较好的稳定性和可靠性，所检测出的肿瘤细胞具有活性，而且可进一步通过单细胞测序等技术获得细胞与分子层面的信息。

## 4 CTCs的临床价值

### 4.1 CTCs的筛查诊断

目前，LDCT、肿瘤自身抗体^[56]^、肿瘤标志物等方法在肺癌筛查方面尚不能单独精准地鉴别良恶性，例如最常应用的LDCT，由于肺小结节在影像学呈现出多样性的特点，异病同影/同病异影给鉴别良恶性造成很大困难，从而导致较高的假阳性率，过度诊断，同时也可能造成延误诊断、以及随访复查加剧患者焦虑心理、影响患者正常工作生活^[10]^，此外，它还存在医源辐射、筛查费用高等弊端。理论上讲，早期肺癌患者，甚至在肺内尚未形成微小病灶之前，外周血中已存在CTCs，主要难点在于检测手段是否可以有效地检测出CTCs，即灵敏性、特异性的问题。

一项关于168例慢性阻塞性肺疾病（chronic obstructive pulmonary disease, COPD）患者的研究^[57]^，以每年胸部CT和ISET法检测CTCs为比较，查看患者COPD有无向肺癌转变。结果显示当ISET法检测出5例患者外周血内存在CTCs时，胸部CT检查并未发现肺部小结节，此后随访监测1年-4年，胸部CT才发现肺部小结节并行手术治疗，术后病理均为Ⅰa期肺癌。这项研究显示出液体活检的优势，CTCs的检测可以早于胸部CT发现非常早期的癌，其辅助影像学检查有助于提高诊断的准确率，从而早期治疗，使术后生存获益。

CTCs的检测还有助于鉴别肺结节，尤其是肺小结节的良恶性，包括肺磨玻璃结节（ground-glass nodules, GGNs）。早期国外研究^[58]^采用CellSearch法对150例患者检测其有无CTCs，去区别良恶性，结果发现肺癌患者组CTCs阳性率30.6%，非肺癌组12%，CTCs数量在肺癌组显著高于非肺癌组，但是受试者工作特征曲线（receiver operating characteristic curve, ROC曲线）分析CTCs检测并无充分的能力去鉴别良恶性。与前期研究不同，Chen等^[59]^的研究中，肺癌组与肺部良性疾病、健康者对照组对比表明前者CTCs阳性率84%，后者仅4.2%。国内学者^[17, 55]^采用靶向PCR CTC检测技术检测非小细胞肺癌患者（non-small cell lung cancer, NSCLC）与肺部良性疾病患者、健康受试者外周血中有无CTCs，发现肺癌患者与非肺癌患者外周血中CTCs存在显著差异（*P* < 0.001），虽然检出率受分期影响^[60]^，但是Ⅰ期NSCLC患者的诊断灵敏性达到67.2%。因此，随着新技术的开发改进，CTCs的检测联合CT检查有望弥补单纯CT检查在肺部疾病良恶性鉴别中的不足，此外，亦可避免患者接受胸部CT引导下穿刺等有创检查带来的风险。

### 4.2 CTCs的疗效评估

早、中期肺癌最有效的治疗手段首选手术根治，术后依据病理结果决定是否辅以放、化疗、靶向治疗以及中医药等其他治疗。但是，无论分期多少，肺癌患者手术后常常死于复发或转移。形成这种结局的原因^[61]^首先的可能是术前已存在未发现的微转移病灶或者持续性的局部病变，其次的可能是治疗本身例如手术、活检等与病灶接触性的操作，在某时某种条件下可能引发或促进了CTCs向循环血液中播散，为以后转移埋下了隐患。2000年相关研究^[62]^结论已表明相比较于开胸肺叶切除手术，电视辅助胸腔镜手术（video-assisted thoracic surgery, VATS）在NSCLC患者手术治疗中可能会增加肿瘤细胞播散进入循环血液的风险。这种推测原因首先是肺部病变不能直观可视，需要使用器械或者手指触摸确定病灶的存在及其位置；其次是因为手术过程中有可能频繁牵拉病肺，以及其他未知欠规范的操作和主观因素。虽然入血的CTCs不一定存活、形成转移灶，但是增加了复发转移的风险，并且预示着预后不良^[63]^，这也很有可能是少部分早期肺癌患者术后早期复发转移的原因之一。

毋庸置疑，VATS是当今胸外科微创手术的主流，尤其在肺癌治疗方面，优势显著，是最佳选择的治疗手段。但是，如果确实手术中存在一些促使肿瘤细胞入血的因素，那么这些因素应该及时被发现、被理解，并且应当受到积极面对，条件允许的情况下尽量避免，从而完善手术操作，获得更好的疗效。

对于晚期肺癌患者，姑息性治疗缓解严重的症状是最主要的。一项纳入了20例晚期肺癌患者，并经过支气管镜下冷冻治疗解除气道肿瘤阻塞的研究^[64]^表明，相比较于治疗前的CTCs，治疗后75%的患者CTCs增加，在一些患者中增加尤为明显，因此，治疗同时也有可能促进CTCs扩散，形成转移。

大量研究已显示CTCs的检测还可以评估化疗方案的可行性和疗效的优劣，从而指导化疗方案的调整。例如Gorges等^[65]^研究，采用一种新的体内装置捕获CTCs，对比分析含铂双药化疗前后患者的CTCs数量变化，结果发现部分缓解（partial response, PR）/完全缓解（complete response, CR）的患者CTCs数量平均下降8.25个，稳定（stable disease, SD）患者无变化，而病情进展（progressive disease, PD）患者平均增加了4.5个，说明CTCs数量的变化可以反映化疗的疗效。Zhao等^[66]^采用微流控技术，选择多种核酸适配体去捕获CTCs，结果显示可以实时地反应肿瘤的异质性，获取比较全面的信息，监测患者对化疗敏感性，评估疗效，指导治疗。此外，CTCs的检测也可以分析肺癌患者有无*EGFR*突变^[67]^、*ALK*重排^[68]^，指导个体化靶向治疗以及评估靶向治疗的耐药情况。

### 4.3 CTCs的术后监测与预后判断

手术治疗后的肺癌患者或者系统化治疗的肺癌患者，均需要定期复查，监测有无复发或转移。相比较常规胸部CT检查，CTCs的检测可以更早地更准确地反应体内肿瘤活动的情况，因此对术后发现早期复发转移的意义重大。若在手术后早期检测出多量CTCs，则有可能表明体内残留肿瘤细胞过多，结合病理情况制定个体化治疗方案可能有益于患者术后长期生存。

目前，临床上主要依据术后TNM分期、病理类型、肿瘤标志物等来判断预后，CTCs检测在判断预后方面具有灵活性、准确性的特点，如果与前者结合起来判断会更加科学合理。Chen等^[69]^以CEA联合检测外周血中CTCs来判断NSCLC患者的预后，结果提示两者呈正比关系，两者均高的患者预后更差。有研究^[70]^采用CellSearch法和ISET法对将行手术根治的NSCLC患者在术前检测CTCs，结果显示有CTCs的患者的无疾病生存期（disease-free survival, DFS）比无CTCs的患者的DFS要差，并提出术前CTCs的检测可以成为判断术后预后的重要标志物。一项纳入20项研究*meta*分析^[71]^表明CTCs数量和肿瘤分期、淋巴结转移有关，和肿瘤类型无关。此外，NSCLC患者中CTCs的存在预示着比较差的总生存时间（overall survival, OS）与无进展生存时间（progression-free survival, PFS）。总之，以上研究均表明，CTCs的检测有望成为临床上判断肺癌预后的有力工具。

## 5 CTCs检测的优缺点

CTCs和循环肿瘤DNA（circulating tumor DNA, ctDNA）是液体活检最主要的部分，也均为目前研究的热点。ctDNA是指体内的肿瘤细胞凋亡、坏死后释放入循环血液的DNA片段^[29]^。相比较于ctDNA，CTCs包括了DNA、RNA、蛋白质等方面的信息，能够更全面的反应肿瘤，但是在反映肿瘤异质性方面可能逊色于ctDNA。检测方面，它们均有无创、便捷、可重复性好的优点，但是检出率低，检测的灵敏性、特异性低成为限制其发展的瓶颈，究其原因，首先和检测技术有关；其次和肿瘤类型、肿瘤大小、负荷有关；最后和CTCs、ctDNA的半衰期有关。例如，大量CTCs从原发肿瘤部位进入循环血液，到达外周血，经过毛细血管网、淋巴结、脏器的过滤，再经静脉回到心脏，反复循环过程中，最后仅仅极少部分（0.02%）可以存活^[61]^。

## 6 小结

肺癌的早期诊断、早期治疗是提高术后生存率的关键，但是现如今单纯依据LDCT对早期肺癌的筛查还具有一些问题，利弊还存在争议，其中最主要的问题是它对大部分肺小结节不能及时有效地鉴别出良恶性。外科手术是早期肺癌最优治疗手段，近20年来手术技术日臻成熟，电视辅助胸腔镜手术（video-assisted thoracic surgery, VATS）下肺叶切除、VATS下精准肺段切除、VATS下解剖性部分肺叶切除与VATS下支气管袖式肺叶切除等术式精益求精，但是患者仍面临5年生存率较低的问题，尽管是早期肺癌。这一原因值得去探讨，去分析研究。

CTCs作为原发肿瘤的一部分，参与其发生、发展、转移整个演变过程，并且携带其丰富的生物学信息。目前，CTCs进入循环血液后的存活机制、转移机制尚不清楚，这将可能成为以后研究的重要方向。此外，目前CTCs的检测以采集外周血，体外检测为主。这种以“部分血液”的检测检出率极低，而且CTCs入循环血液后大量被分散、被破坏。为了避免这些不足，CTCs的检测可能会从体外检测向体内检测发展，比如发明捕获CTCs的滤网装置，通过血管介入将其置入到实体瘤器官的主要静脉，并可以在体外装置感应其捕获的情况。随着CTCs检测技术的改良进步，它将可能在肺癌患者全程治疗过程中扮演重要角色，尤其是对早期肺癌的诊断与治疗。
